# Neurodegeneration and Epilepsy in a Zebrafish Model of CLN3 Disease (Batten Disease)

**DOI:** 10.1371/journal.pone.0157365

**Published:** 2016-06-21

**Authors:** Kim Wager, Anselm A. Zdebik, Sonia Fu, Jonathan D. Cooper, Robert J. Harvey, Claire Russell

**Affiliations:** 1 Department of Comparative Biomedical Sciences, Royal Veterinary College, Royal College Street, London, NW1 0TU, United Kingdom; 2 Department of Neuroscience, Physiology and Pharmacology, UCL Medical School, Royal Free Campus, Rowland Hill Street, London, NW3 2PF, United Kingdom; 3 Department of Nephrology, UCL Medical School, Royal Free Campus, Rowland Hill Street, London, NW3 2PF, United Kingdom; 4 Pediatric Storage Disorders Laboratory, Department of Basic and Clinical Neuroscience, Maurice Wohl Clinical Neuroscience Institute, Institute of Psychiatry, Psychology & Neuroscience, King’s College London, 5 Cutcombe Road, London, SE5 9RX, United Kingdom; 5 Department of Pharmacology, UCL School of Pharmacy, 29-39 Brunswick Square, London, WC1N 1AX, United Kingdom; Institut Curie, FRANCE

## Abstract

The neuronal ceroid lipofuscinoses are a group of lysosomal storage disorders that comprise the most common, genetically heterogeneous, fatal neurodegenerative disorders of children. They are characterised by childhood onset, visual failure, epileptic seizures, psychomotor retardation and dementia. CLN3 disease, also known as Batten disease, is caused by autosomal recessive mutations in the *CLN3* gene, 80–85% of which are a ~1 kb deletion. Currently no treatments exist, and after much suffering, the disease inevitably results in premature death. The aim of this study was to generate a zebrafish model of CLN3 disease using antisense morpholino injection, and characterise the pathological and functional consequences of Cln3 deficiency, thereby providing a tool for future drug discovery. The model was shown to faithfully recapitulate the pathological signs of CLN3 disease, including reduced survival, neuronal loss, retinopathy, axonopathy, loss of motor function, lysosomal storage of subunit c of mitochondrial ATP synthase, and epileptic seizures, albeit with an earlier onset and faster progression than the human disease. Our study provides proof of principle that the advantages of the zebrafish over other model systems can be utilised to further our understanding of the pathogenesis of CLN3 disease and accelerate drug discovery.

## Introduction

Lysosomal Storage Disorders (LSDs) represent a group of 60 or more predominantly recessively inherited monogenic disorders, the majority of which cause neurodegeneration (Parenti *et al*., 2015). The neuronal ceroid lipofuscinoses (NCLs) or Batten disease are a group of LSDs that comprise the most common, genetically heterogeneous, fatal neurodegenerative disorders of children [1], with rare adult forms. The NCLs are characterised by variable age of onset, visual failure, epileptic seizures, psychomotor retardation and dementia. Collectively, the NCLs affect ~14 000 people worldwide [2]. To date, 13 genetic loci have been identified as causing NCL and their classification is based upon the mutated gene and age of onset [3]. Each gene is called CLN followed by a designated number, where CLN stands for ceroid-lipofuscinosis, neuronal. For example when *CLN3* is mutated, the most common onset is juvenile, so is referred to as juvenile CLN3 disease. The incidence of CLN3 disease, the focus of this study, ranges from 0.25–7 per 100 000 in Europe [2].

The pathological hallmark of NCLs is storage material in lysosomes, but the content and morphology of these storage vesicles vary. In CLN3 disease, subunit c of mitochondrial ATP synthase (subunit c) is a major component of the storage material [4] but it is not known if accumulation of storage material is protective or pathological, or if subunit c is a specific component within the pathway in which CLN3 protein functions [5]; [4]; [6]. Indeed, very little is known about the expression and function of the CLN3 protein (reviewed in [6] and [7]). CLN3 is a transmembrane protein, possibly ubiquitous throughout all tissues, and localised primarily to the endosome and lysosome. The CLN3 protein has been linked to trafficking, autophagy, osmoregulation, cell migration, cell morphology, proliferation and cell survival, but the primary function of this protein is unclear [6]; [7].

The presenting symptom in CLN3 disease is a loss of visual acuity, usually between 4 and 7 years old [2], leading to blindness within 2 to 10 years [8]. Cognitive decline, behavioural and psychiatric problems follow. Epileptic seizures develop between 7 to 18 years and are predominantly of the tonic-clonic type, although partial complex and myoclonic seizures are also observed. Seizures worsen and may frequently be, or become over time, refractory to medication [9]; [10]. Around puberty, motor symptoms evolve, manifesting primarily with extrapyramidal and cerebellar signs including hypokinesia, muscle rigidity, altered gait and imbalance, resembling a parkinsonian phenotype [11]; [8]; [12]. Psychiatric problems include hallucinations, depression, dementia and insomnia [13]. In addition to the neurological features, a cardiac abnormality develops in the second decade of life [14]; [9], and the immune system is compromised [15].

Symptoms are accompanied by a progressive reduction in brain weight (mainly attributed to cerebral cortical atrophy) and white matter, enlargement of cerebrospinal fluid spaces, and cerebellar atrophy [16]. Neuron loss is, however, selective. The substantia nigra often lacks pigment (which may explain the Parkinsonism features) and there is loss of Purkinje cells and granular cells in the cerebellum, and of granular cells in the dentate nucleus [5]. In addition, neuronal loss and glial activation has been noted in the hippocampus [17]. However, detailed quantitative information about neuropathological changes in human CLN3 disease is limited, with qualitative studies restricted to autopsy material.

Mouse models have proved invaluable for studying the progression of CLN3 disease. Each of the four genetic models recapitulates many features of the human disease [7]. Although there are subtle mutation and strain dependent phenotypic differences in age of onset and severity, all show a progressive onset of neurological deficits affecting vision, learning and memory and motor function. These are associated with neuron loss in the retina, thalamus, cortex, cerebellum, substantia nigra and striatum, which is preceded by glial activation [18]; [7]; [19].

Treatments for CLN3 disease are palliative, relying largely on anti-epileptic drugs, which have limited prolonged efficacy in this disorder [2], and _L_-DOPA [9]. Indeed, no disease modifying therapy for CLN3 disease exists. Enzyme replacement therapy (ERT), gene therapy and stem cell therapy, aimed at correcting for deficient proteins, are potential disease modifying treatments for other forms of NCL [20]. However, as CLN3 disease is caused by mutations in a transmembrane protein of unknown function that is not normally trafficked out of cells, between cells or taken up from the extracellular space into cells, it is highly improbable that protein delivered to the cerebrospinal fluid would be taken up by cells. Furthermore, introduction of functional protein by gene or stem cell therapy would need to be more widespread than is currently possible. In contrast, NCLs caused by mutations in lysosomal enzymes such as TPP1 (causing CLN2 disease) are amenable to ERT, due to the ability of the protein to cross-correct (traffic to the extracellular space and then be taken up by other cells), and interim results from the CLN2 disease ERT trial, in which recombinant protein is delivered to the CSF, look promising (Clincaltrials.gov identifier NCT01907087; http://investors.bmrn.com/releasedetail.cfm?ReleaseID=958565; [20]. However, small molecule therapy potentially provides a viable avenue for all of the NCLs [20]. To date, trials using mouse models of CLN3 disease have shown some symptomatic improvement after targeting the AMPA receptor with EGIS-8332 [21]; [22] and the NMDA receptor with memantine [23]. Also promising is a decrease in circulating autoantibodies to brain antigens after immune suppression with mycophenolate mofetil [24], leading to a phase 1 clinical trial of Cellcept (Clincaltrials.gov identifier NCT01399047). Unfortunately, small molecule therapy development for CLN3 disease is severely constrained by our lack of understanding of both the function of the CLN3 protein and the disease pathogenesis. There is also a need for clinically predictive *in vivo* disease models that are amenable to high-throughput small molecule screens.

Zebrafish are small freshwater teleosts that have become a valuable tool for disease modelling, particularly for neurodegeneration [25] and metabolic disease [26]. Tiny, transparent zebrafish embryos and larvae exhibit rapid, oviparous development in high numbers making them an excellent vertebrate tool to house in 96-well plates to facilitate *in vivo* high-throughput screening and development of compounds using relevant, often automatable phenotypes such as locomotion, or the monitoring of fluorescent reporters [27]; [26]; [25]; [28]. Indeed, we have shown that automated locomotion assays reflect changes in motor function in the zebrafish model of CLN2 disease (*tpp1*^*sa0011*^ mutant) [29] and we are currently exploiting these assays for compound testing. Importantly, this method does not require knowledge of the normal or abnormal function of the protein mutated in the disease, nor of the pathogenesis, making this approach particularly suitable for CLN3 disease if a suitable zebrafish disease model was available.

In the present study, we demonstrate that knockdown of *cln3* in zebrafish recapitulates several pathological features of the human disease including reduced survival, retinopathy, neurodegeneration, epileptiform activity and enlarged lysosomes with accumulation of subunit c. Furthermore, we have identified phenotypes such as reduced proliferation in the retina and axonal disorganisation. This model of CLN3 disease will enable us to further understand the pathology of CLN3 disease and serve as a platform for compound testing prior to further testing in more expensive and time-consuming mammalian models.

## Materials and Methods

### *In situ* hybridisation

The BC085653 clone containing the full-length zebrafish *cln3* gene was used. Sense and antisense RNA probes were transcribed by first linearising the plasmid with *Not*I and *Eco*RI. *In vitro* transcription was performed with SP6 and T7 RNA polymerases respectively, using a digoxigenin (DIG) labelled mixture (Roche), according to the manufacturer (Roche). *In situ* hybridisation was then performed, documented and manipulated as previously described [30].

### Generation and maintenance of morphant zebrafish

Adult wild-type (WT) *TupLF* strain and transgenic Tg[*HuC*:*GFP*] zebrafish were housed in a multi-rack aquarium system at the Royal Veterinary College. Zebrafish were reared at 28.5 ± 0.5°C on a 14 hour light, 10 hour dark cycle. Local RVC Animal Welfare Ethics Review Board approval was granted and a UK Home Office Project License was in place in accordance with the UK Home Office Animals Scientific Procedures Act (1986).

Following adult breeding, embryos were incubated in the dark at 28°C, in ‘fish water’, containing aquarium water and methylene blue (0.0002%). To inhibit pigment formation, embryos were raised in 0.2 mM 1-phenyl-2-thiourea (PTU, Sigma) in fish water from 24 hours post-fertilization (hpf) whilst still in their chorion. For live imaging of morphology, larvae were anesthetized with MS222 (0.016% w/v).

To generate *cln3* morphant embryos, morpholino (MO) anti-sense oligonucleotides against *cln3* messenger RNA (mRNA) were pressure-injected into one-two cell stage zebrafish embryos using a glass capillary injection needle. MOs (from Gene Tools, USA) targeting the start ATG (*cln3* ATG MO: CATtgcgactttcacaggagaaatg) or splice site (*cln3* SPL MO: cagcaacCTAAACAGAGATAATACA) of *cln3*, and the start site of *p53* (*p53* MO: gcgcCATtgctttgcaagaattg;) [30], as well as a control mismatch MO (*cln3* ATG mismatch MO: CATTcCGAgTTTgACAcGAcAAATG; mismatched nucleotides are shown in lower case) were used. MO sequences were subjected to BLAST searches to confirm specificity.

### Complementary DNA preparation, cloning and sequencing

To confirm mis-splicing by *cln3* SPL MO, cDNA synthesis by reverse transcription of mRNA, PCR, gel electrophoresis and sequencing was performed as previously described [29, 30]. *cln3*-specific PCR primers (Invitrogen) used were (5’ to 3’): *cln3* AF1 (5’UTR forward)-TGAGCGATGATCATACACGA; *cln3* AR1 (exon 3 reverse)- CGCTCAGCATCACCACATA; *cln3* AR2 (exon 15 reverse)- AAGCAATTCCCAGACTGTCC.

### Spontaneous coiling

Groups of 10 embryonic zebrafish still in their chorions were arrayed in a petri dish and mounted on a Nikon SMZ1500 stereomicroscope (Nikon, UK). Three-minute recordings were obtained with a DMK21AF04 camera (The Imaging Source, Germany) at a frame rate of 30 frames per second (fps) and recorded using Media Recorder (Noldus, distributed by Tracksys, UK). Movies were obtained in .avi format and converted to .mp4 using Video Converter Pro (Digiarty software, China). Videos were played back in slow motion using QuickTime Player (Apple) and coiling or tail flicking events for each embryo counted.

### Escape response

Individual larvae were placed in a petri dish and mounted on a Nikon SMZ1500 stereomicroscope. Fish were touched lightly with a pipette tip on the tail as many times as necessary to elicit the escape response. Recordings were obtained with a DMK21AF04 camera at a frame rate of 30 fps and recorded using Media Recorder (Noldus). Movies were obtained as previously described, and single frames extracted with SnapzPro X (Ambrosia, USA) for figure assembly.

### Whole-mount immunofluorescence

PTU-treated larvae were fixed overnight in 4% (w/v) paraformaldehyde (PFA) in 1 x PBS at 4°C. Subsequently larvae were stored in 100% methanol (MeOH) at -30°C. Larvae were rehydrated in a descending MeOH series, rinsed in PBST (PBS, 0.8% Triton X-100) and subjected to proteinase K (15 ng/ml for 1 hour in PBS + 0.1% Triton^™^ X-100). Subsequently, the larvae were fixed for 20 minutes in 4% paraformaldehyde/PBS, washed several times in PBST and then blocked for 1 hour at RT in blocking reagent (PBST, 1% (v/v) DMSO, 10% normal goat serum). Primary antibodies were diluted in incubation buffer (PBST, 1% (v/v) DMSO, 1% normal goat serum) and larvae incubated at 4°C overnight: mouse anti-acetylated α-tubulin (1:1000) (Sigma), rabbit anti-PH3 (1:1000) (Upstate, distributed by EMD Millipore, UK), rabbit anti-subunit c (1:1000) (gift from Dr. J. Tyynelä) [29], mouse anti-LAMP1 (1:250) (Abcam, UK), rabbit anti-GFAP (1:1000) (gift from Sam Nona and John Scholes) [31]. Larvae were rinsed twice in PBST and then incubated four times for 15 minutes in PBST. Antibody blocking was repeated by incubating larvae for 1 hour at RT in blocking reagent before applying the secondary antibody in incubation buffer for incubation at 4°C overnight: goat anti-mouse Alexa 568 (1:200), goat anti-mouse Alexa 546 (1:200), goat anti-rabbit Alexa 546 (1:200), goat anti-rabbit Alexa 488 (1:200), goat anti-mouse Alexa 488 (1:200) (Invitrogen). Larvae were rinsed twice in PBST and then cleared in 70% (v/v) glycerol in PBS. Fluorescent larvae were imaged with a Leica SP5 confocal microscope (Leica, UK). All images for the same antibody were acquired and manipulated in exactly the same way. Cells were quantified using the cell counter plugin for Image J.

### Retinal area

Single zebrafish larvae were mounted on microscope slides and imaged on a Nikon SMZ1500 stereomicroscope. Still images of the retinae were taken using a DMK21AF04 camera and captured using IC Capture (The Imaging Source, Germany). In Image J, the zebrafish retina was encircled using the polygon tool and the area calculated.

### Surface electroencephalogram (EEG)

Following the published protocol [32], zebrafish aged 4 days post-fertilisation (dpf) were incubated in 2 mM Tubocurarine (Sigma, UK) until paralysed (at 5–20 minutes), rinsed and mounted in 15 g/L type VII low melting point agarose (Sigma) in aquarium water for up to 1 hour EEG recording. They were terminated if the brain showed any opacity or poor perfusion, there was considerable slowing of the heart rate, or if fish started to move. Prior to and during all EEG recordings, all larvae were checked for good peripheral and cerebral blood circulation and monitored to verify paralysis. Microelectrodes were pulled on a Zeitz puller (Zeitz, Germany) from thin-walled borosilicate with filament (GC150 TF-7.5, Harvard Apparatus, UK), manually broken and fire polished to a diameter of 10–15 μm. Electrodes were back-filled with 1 M NaCl, connected to a chlorinated silver wire and placed against the zebrafish head, above the optic tectum. The potential difference between the recording electrode and a reference electrode placed in the agarose close to the zebrafish was amplified 10,000× with a DAGAN 2400 amplifier (Dagan, USA), band pass filtered at 0.3–300 Hz and EEGs digitized at 2 kHz via a PCI-6251 interface (National Instruments, UK) connected to a custom-made front end using WinEDR V.3.0.8 software (John Dempster, University of Strathclyde, UK).

Regions of 20 seconds duration spanning spiking, or in the case of WT, baseline activity, were extracted using WinEDR v.3.0.8, and the numerical values imported into Origin 7 (OriginLab, USA) and subjected to a Fast Fourier Transformation (FFT) to generate the frequency spectrum for the region of interest. Five regions of interest were extracted per zebrafish EEG and the resulting FFTs averaged to produce an average frequency spectrum for the fish in question. These averaged FFTs were then further combined with other fish of the same treatment group to produce an overall average spectrum. This frequency spectrum was then plotted against amplitude to reveal the averaged spectral composition of the recorded EEG signals in the frequency domain.

### Vital stains

To visualise apoptotic cells, lysosomes and mitochondria, *in vivo* staining was carried out in live, PTU-treated, manually-dechorionated, larvae with the vital dyes acridine orange (AO; Sigma-Aldrich), LysoTracker red (LTR; Invitrogen) and Mitotracker red (MTR; Invitrogen), respectively. Larvae were incubated at RT, in either 1.35 μM AO and 10 μM LTR, or 10 μM MTR in PBS for thirty minutes on a rocker platform, protected from light. Larvae were rinsed twice in PBS, mounted in 2% (w/v) low melting point agarose (Sigma) in aquarium water and viewed with a Leica SP5 confocal microscope. All images for the same stain were acquired and manipulated in exactly the same way.

### Statistics

Statistical analyses were performed using Prism 6 (GraphPad software, USA). Specific tests for each experiment are stated in the results. Statistical significance for all tests was referred to as *p*≤0.05 *, *p*≤0.01 **, *p*≤0.001 ***, *p*≤0.0001 ****.

## Results

### Zebrafish *cln3* is expressed in the developing zebrafish

**Fig 1:**
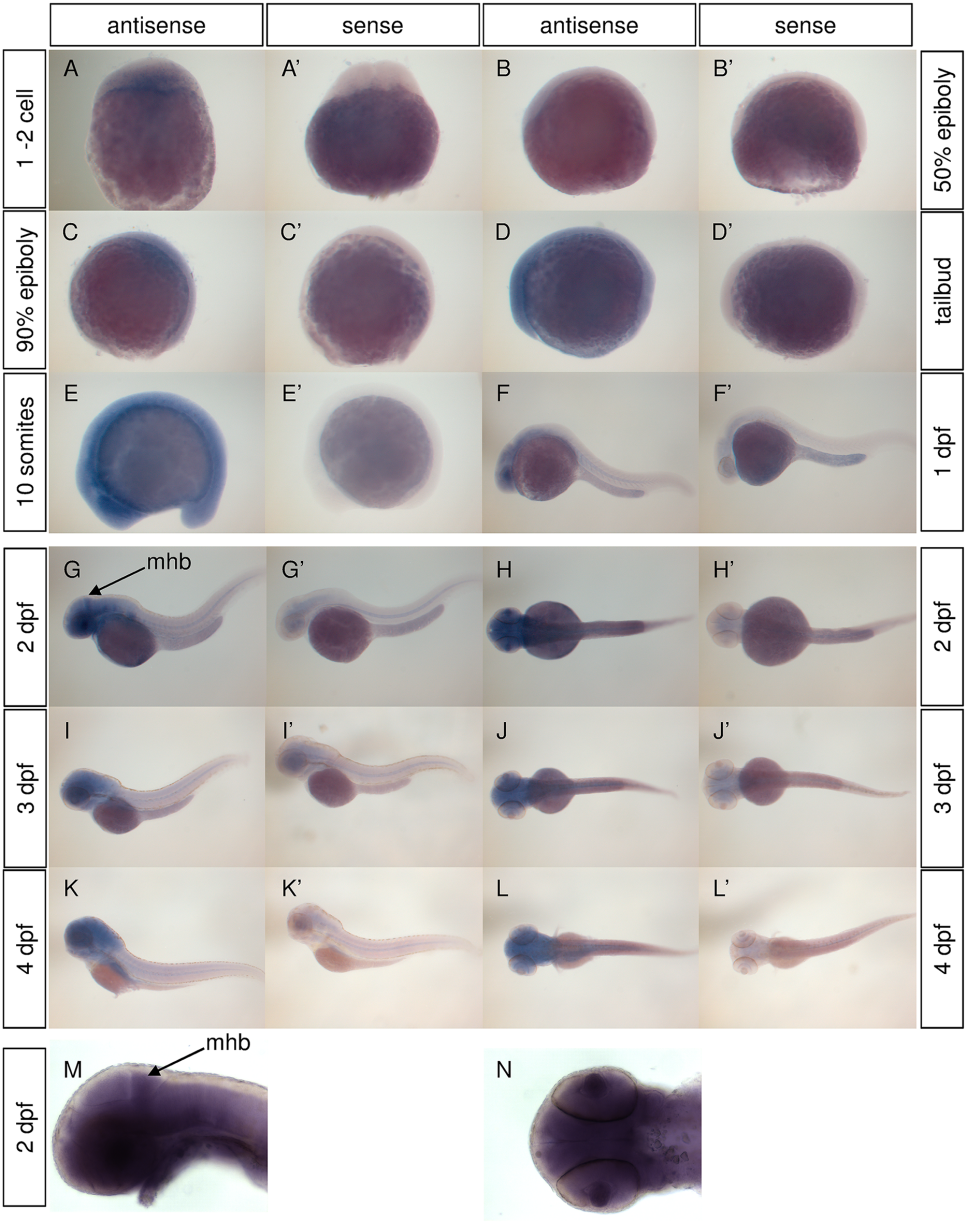
*cln3* is expressed in the developing WT zebrafish. A-N) *In situ* hybridisation for *cln3* (antisense) shows its expression from the one cell stage through to 4 dpf. From 1 dpf expression is increasingly restricted to the central nervous system. At 2 dpf *cln3* expression is particularly strong in the mid-hindbrain boundary (arrow) where the cerebellum is developing (G, H, M, N). (A’-L’) Experiments conducted using a *cln3* sense probe (sense) revealed the specificity of the antisense signal. Abbreviations: mhb, mid-hindbrain boundary.

*cln3* gene expression was characterised in WT zebrafish using *in situ* hybridisation (Fig 1). *cln3* expression was detected in newly fertilised WT embryos (Fig 1A), demonstrating deposition of *cln3* mRNA into the egg by the mother, and is ubiquitously expressed until the 20 somite stage. Subsequently, expression was observed in the retina and throughout the central nervous system until 4 dpf. At 2 dpf expression was particularly strong in the prospective cerebellum (Fig 1G and 1M). These data confirm that *cln3* is expressed in development and could potentially give an embryonic or larval phenotype when inactivated by gene knockout.

### Protein conservation and efficacy of antisense morpholino knockdown of *cln3*

**Fig 2:**
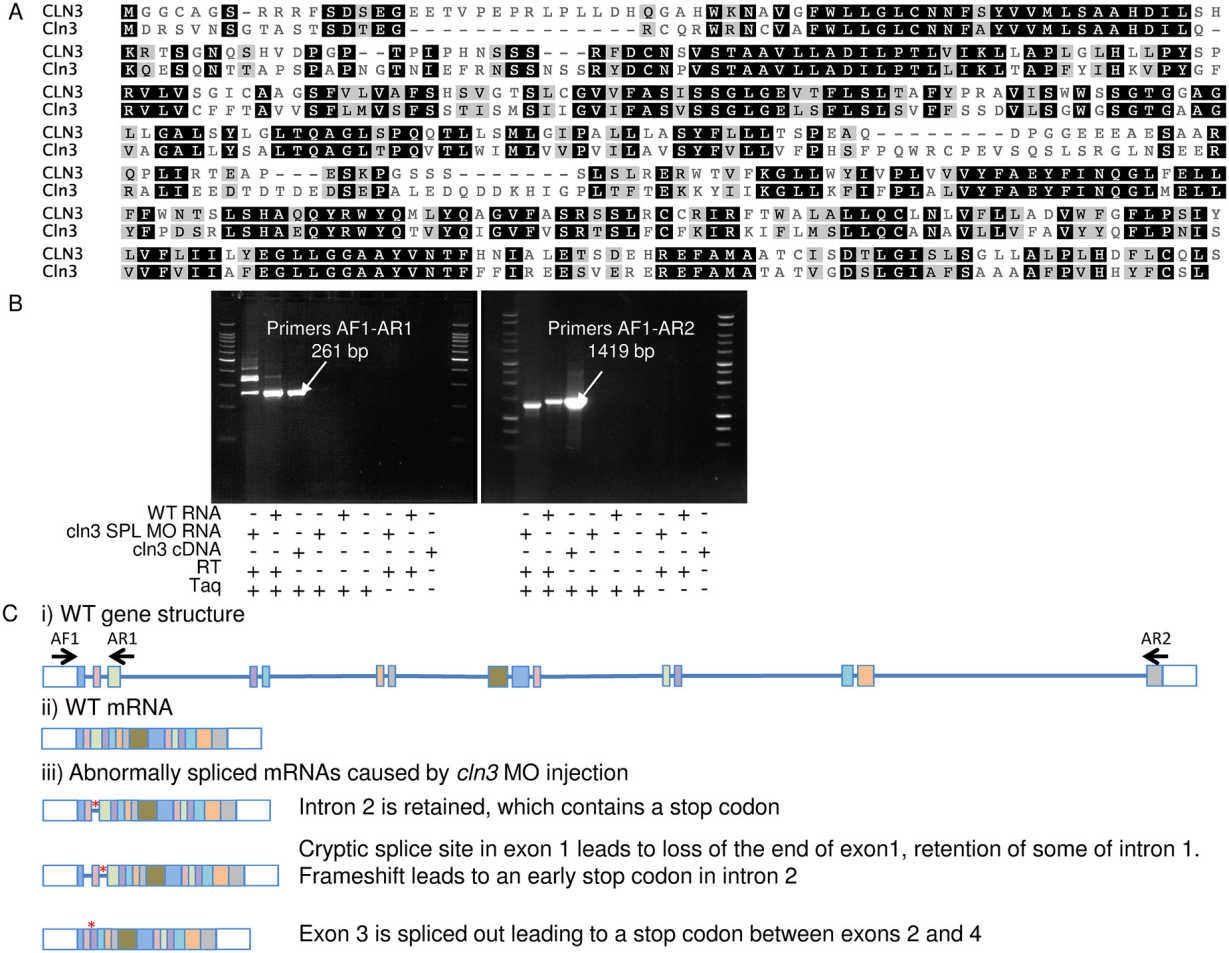
Identification and morpholino targeting of the zebrafish Cln3 gene. (A) CLUSTAL alignment indicates that zebrafish Cln3 amino acid sequence (second row) is 62% similar and 49% identical to human CLN3 (first row). Black background indicates identical residues shared between human and zebrafish, grey background indicates similarity, ‘-’ indicates a gap in the alignment. (B) RT-PCR using primer pairs AF1-AR1 and AF1-AR2 (see C), followed by sequencing, confirmed that the *cln3* SPL MO affected mRNA splicing. Lanes one and two show the PCR product from cDNA made from *cln3* SPL MO injected embryos and WT embryos respectively. Arrow in lane three points to the positive control, which is the PCR product generated using plasmid containing *cln3* mRNA as a template. (C) i) the gene structure of WT *cln3* showing the relative position of the PCR primers used. ii) The WT mature mRNA. iii) Splice variants caused by injection of *cln3* SPL MO. Key: lines, introns; boxes, exons; white boxes, untranslated regions; red asterisk, stop codon.

CLUSTAL alignment showed that zebrafish Cln3 protein (446 amino acids) (GI:55925536) shares an amino acid sequence similarity of 62% and identity of 49% with human CLN3 protein (438 amino acids) (GI:5801849) (Fig 2A). Given this level of conservation, we hypothesised that loss of this protein in zebrafish is likely to have phenotypic consequences relevant to human CLN3 disease.

*cln3* morphant zebrafish were generated by injecting MOs against *cln3* into WT embryos. No sufficiently specific antibody recognises endogenously expressed zebrafish Cln3 so we were unable to confirm reduced translation and loss of Cln3 protein after *cln3* ATG MO injection. To confirm that *cln3* SPL MO affected splicing of the *cln3* gene, RT-PCR and sequencing was conducted on WT and morphant larvae (Fig 2B). This analysis showed that *cln3* SPL MO caused aberrant splicing, producing three novel mRNA isoforms, each resulting in a stop codon after exon two and is therefore predicted to produce a short protein that is unlikely to have full function (Fig 2C).

### Both *cln3* morphants exhibit abnormal morphological characteristics consistent with neurodegeneration

**Fig 3:**
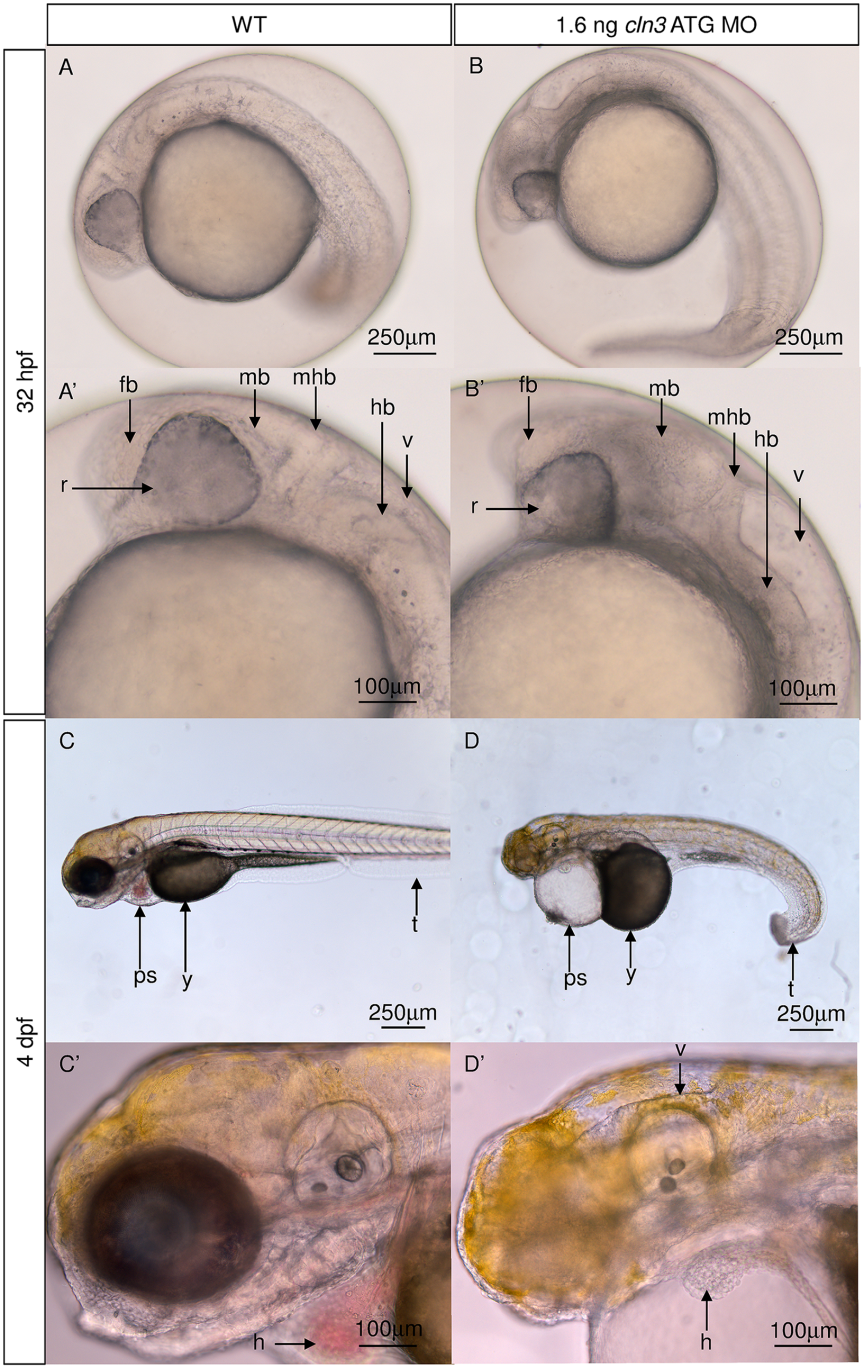
*cln3* ATG MO morphants have abnormal brain and heart morphology. (A, A’) 32 hpf normal development of the WT forebrain, midbrain, hindbrain, retina and fourth ventricle. (B, B’) 32 hpf 1.6 ng *cln3* ATG MO morphants display abnormal development of all parts of the brain, a smaller retina, and enlargement of the fourth ventricle. (C, C’) normal development of the WT tail, yolk, pericardial sac and heart at 4 dpf. The WT brain completely fills the cranium. (D, D’) 1.6 ng *cln3* ATG MO morphants have a curved tail, and a larger yolk and pericardial sac at 4 dpf. The heart has an elongated appearance and is lacking pigmented erythrocytes. The fourth ventricle is enlarged and the mid- and hindbrain appear smaller. Lateral views. Anterior is to left. Dorsal is up. Abbreviations: v, fourth ventricle; hb, hindbrain; mhb, mid-hindbrain boundary; mb, midbrain, fb, forebrain, r, retina; t, tail; y, yolk; ps, pericardial sac; h, heart; (*n* = 4 per group). Lateral views. Scale bars: A-D 250 μm; A’-D’ 100 μm.

In human juvenile CLN3 disease, neuroimaging reveals ventricular dilation with atrophy of the cerebral cortex, particularly the occipital lobes, and cerebellum [16]. In addition human patients display progressive morphological and functional cardiac abnormalities [14]. Bright-field microscopy revealed that the morphology of the *cln3* ATG MO morphant brain at 4 dpf is grossly abnormal, with changes noticeable from 32 hpf (Fig 3). At 4 dpf we observed a large decrease in the size of the midbrain and hindbrain, the retina appeared smaller, the fourth ventricle appeared dilated and the morphant heart lacked pigmented erythrocytes and had an elongated appearance (Fig 3D and 3D’).

Further bright-field imaging was conducted at the earlier age of 32 hpf to observe how these phenotypes progressed over time. At this earlier age, *cln3* ATG MO morphants already displayed abnormal development of the midbrain tectum, midbrain hindbrain boundary, and reduced caudal hindbrain with enlargement of the fourth ventricle (Fig 3B and 3B’). At this stage the morphant retinae also already appeared smaller and greyed, suggestive of cell death. These findings are not only relevant to the human disease, but highlight the potential for such morphants to reveal disease-related phenotypes.

### Higher specificity and minimal toxicity from the *cln3* ATG morpholino

We compared *cln3* ATG MO and *cln3* SPL MO morphants at a variety of doses using bright-field microscopy. A higher dose of *cln3* SPL MO (12 ng) was needed to cause the same morphant phenotype as that caused by *cln3* ATG MO (1.6 ng) at 4 dpf (S1 Fig). This is likely because *cln3* SPL MO disrupts splicing and therefore does not affect the *cln3* mRNA deposited by the mother, whereas the *cln3* ATG MO disrupts translation of both maternal and zygotic *cln3* mRNA. Both morphants manifested small retinae, reduced brain size, pericardial oedema, failure of the swim bladder to inflate, lack of yolk resorption and a curved tail (S1 Fig).

**Fig 4:**
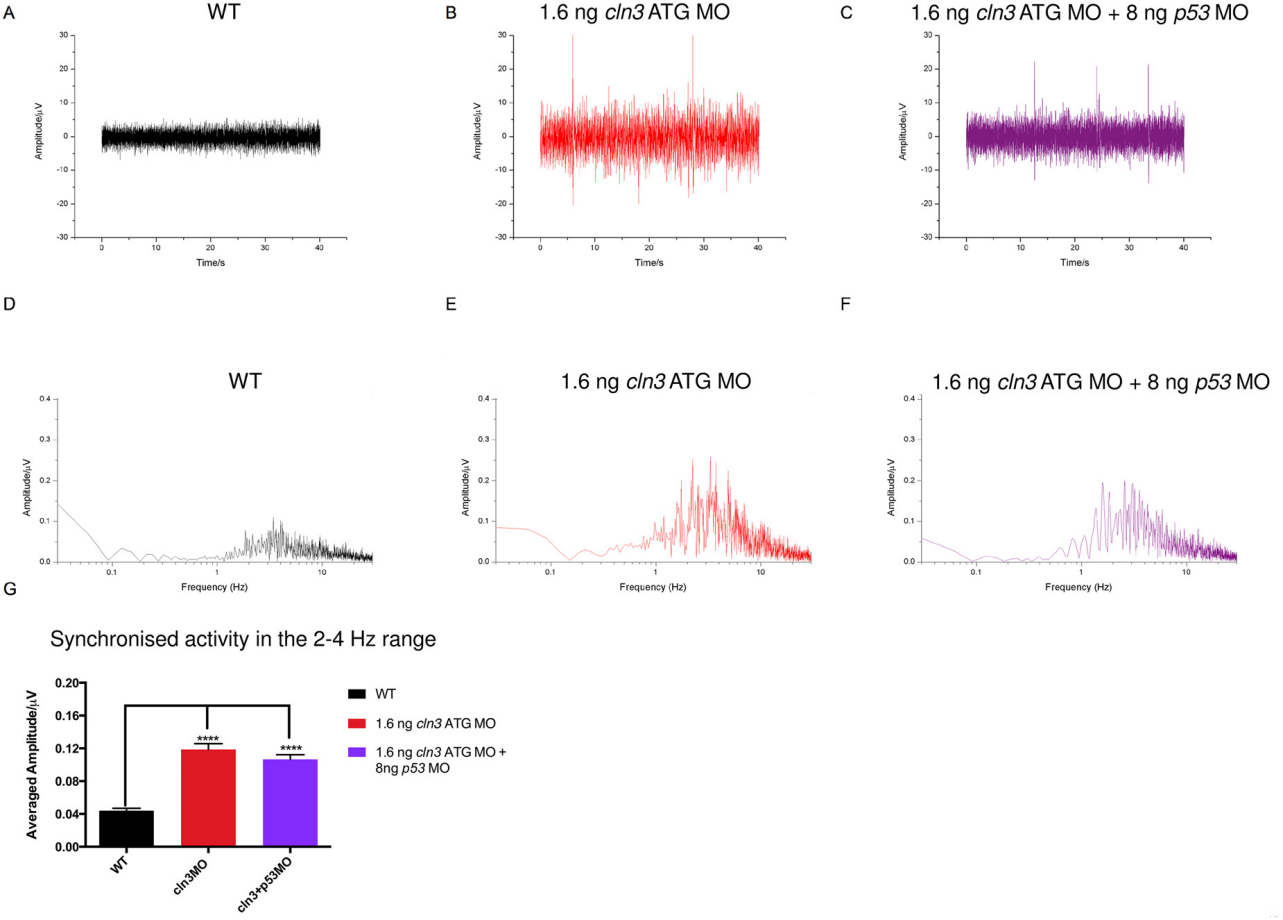
*cln3* ATG MO morphants display epileptiform activity in the 2–4 Hz range. Single channel surface EEG shows frequent high amplitude spiking in both 1.6 ng *cln3* ATG MO morphants (B) and 1.6 ng *cln3* ATG MO + 8 ng *p53* MO double morphants (C) compared to WT (A). Fast Fourier transformation (FFT) was performed on 20 s representative samples, 5 times per trace and an average produced per trace; an average FFT per genotype was then used to calculate the mean frequency spectrum. The greatest spiking amplitude was observed between 2–4 Hz in the 1.6 ng *cln3* ATG morphants (E) and 1.6 ng *cln3* ATG MO + 8 ng *p53* MO double morphants (F) compared to WT (D), and this was a statistically significant difference (G). WT *n* = 5, *cln3* ATG MO *n* = 6, *cln3* ATG MO + *p53* MO n = 6.

We reasoned that using a lower dose of MO is less likely to cause toxicity so all further experiments were performed with the *cln3* ATG MO. To confirm that the observed phenotype was not caused either by off-target effects or general MO toxicity, we injected a 5 bp mismatch MO (1.6 ng *cln3* ATG mismatch MO) and a MO targeting *p53* (8 ng *p53* MO) and found that neither produced a morphological phenotype (data not shown). *p53* MOs are frequently used because MO toxicity, if it occurs, is caused by activation of *p53* which leads to apoptosis [33]. Co-injection of 8 ng *p53* MO with 1.6 ng *cln3* ATG MO did not rescue or reduce the *cln3* ATG MO morphological (data not shown) or electroencephalography (EEG) phenotype (Fig 4), indicating that the *cln3* ATG MO did not cause p53-mediated toxicity. Hence, for all subsequent experiments the *cln3* ATG MO was used and uninjected WT fish used as controls unless otherwise stated.

### *cln3* morphant EEGs show epileptiform activity

Surface EEG recording from the optic tectum [32] was used to investigate the possible presence of epileptiform activity in 4 dpf *cln3* ATG MO morphants. EEGs recorded from WT, *cln3* ATG MO morphants and *cln3* ATG MO (1.6 ng) + *p53* MO (8 ng) morphants (Fig 4A–4C) were analysed by Fast Fourier Transformation (FFT) and the frequency spectrum was plotted against amplitude to reveal the strength of the various frequency components in the original EEG signal (Fig 4D–4F). This revealed increased activity at a frequency of 2–4 Hz (Fig 4E and 4F), the amplitude of which was significantly higher in both *cln3* ATG MO morphants and those co-injected with *p53* MO (Fig 4G) (*p*<0.0001). This indicates synchronised neuronal activity within this frequency range and reflects epileptiform activity.

### *cln3* morphant survival is impaired

**Fig 5:**
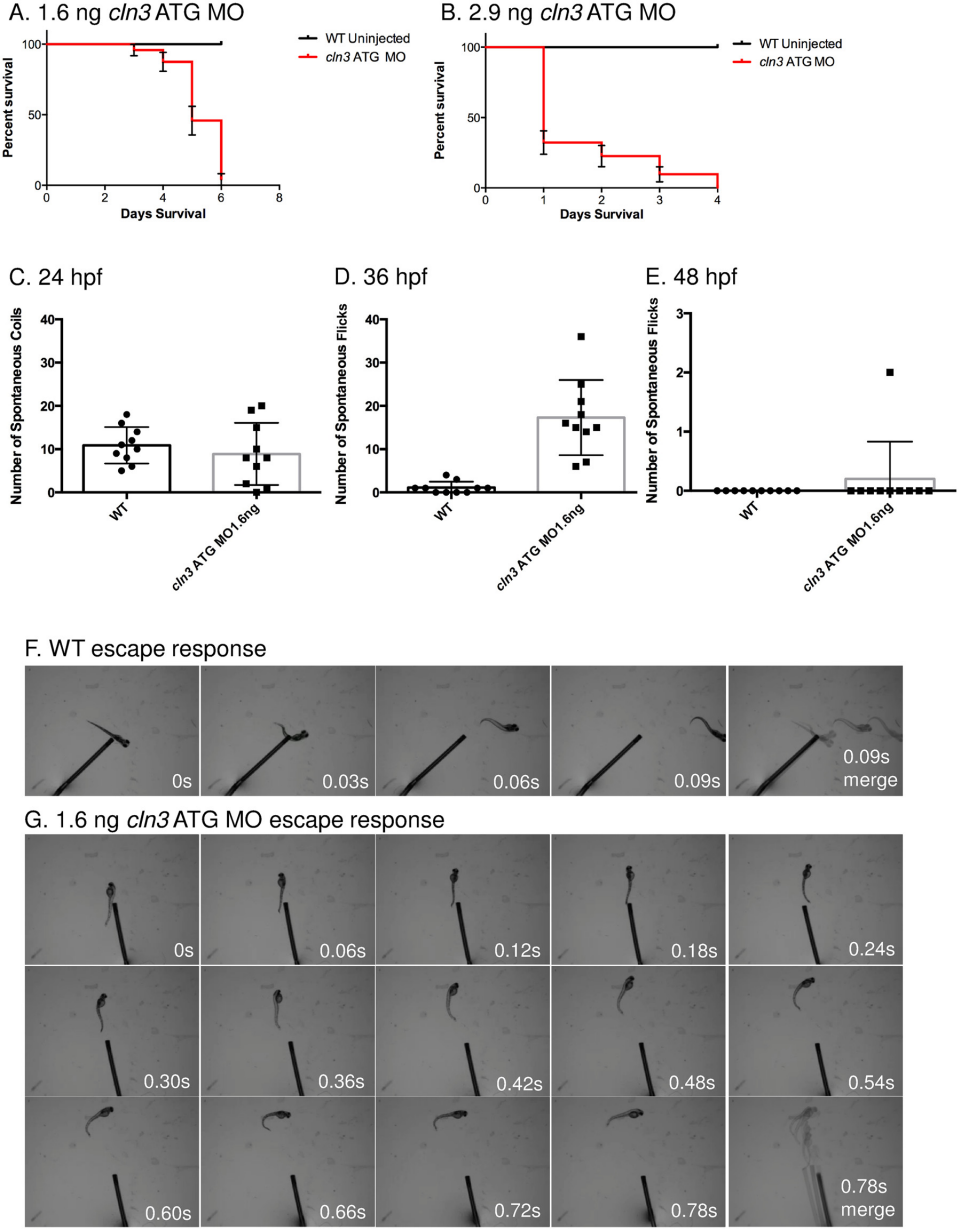
Survival and activity are compromised in *cln3* ATG MO morphant zebrafish. (A-B) *cln3* ATG MO morphants die prematurely. Progression of *cln3* ATG morphants demonstrated by monitoring cohorts of fish injected with 1.6 ng or 2.9 ng of *cln3* ATG MO and uninjected WT siblings. (A) The Kaplan-Meier survival curve shows that all WT larvae survive beyond 6 dpf, whereas the majority of morphants injected with 1.6 ng *cln3* ATG MO die between 3 and 6 dpf (median survival 5 dpf). Log rank (Mantel-Cox) test *p*<0.0001 (*n* = 46 WT and *n* = 23 morphants). (B) The Kaplan-Meier survival curve shows that all WT larvae survive beyond 4 dpf, whereas the morphants injected with 2.9 ng *cln3* ATG MO die between 1 and 4 dpf (median survival 1 dpf). Log rank (Mantel-Cox) test *p*<0.0001 (*n* = 16 WT and *n* = 31 morphants). Error bars indicate ± SE. (C-E) Increased activity was observed in *cln3* ATG MO morphants at 36 hpf. Analysis of number of spontaneous coils or tail flicks within a 3 minute time period comparing 1.6 ng *cln3* ATG MO morphants with age matched WT siblings was carried out at 24 hpf (C), 36 hpf (D) and 48 hpf (E). At 24 and 48 hpf the data show no significant difference in the number of coils or flicks. At 36 hpf the data show a significant difference in the number of flicks; *p*<0.0001 (*n* = 10 zebrafish per treatment group). Data represent mean ± SD; results were evaluated using a 2-tailed unpaired Student’s *t*-test. (F-G) The *cln3* ATG MO morphant escape response is diminished. (F) The WT (control) fish aged 4 dpf respond to touch with a C-bend, a turn away from the stimulus and rapid swimming (0.03 second intervals). (G) 1.6 ng c*ln3* ATG MO morphants aged 4 dpf display a greatly attenuated escape response (0.06 second intervals).

Human patients suffering from juvenile CLN3 disease die in the third or fourth decades of life [12]. To examine the survival of *cln3* ATG MO morphants, a Kaplan-Meier survival analysis was performed revealing that *cln3* ATG MO morphants (1.6 ng) had a median survival of 5 dpf with a range of 1–6 dpf. In contrast, no WT larvae died within the time frame studied (Fig 5A). Zebrafish injected with 2.9 ng of *cln3* ATG MO had a median survival of 1 dpf with a range of 1–4 dpf (Fig 5B). The log rank (Mantel-Cox) analysis to test the null hypothesis of no difference in survival functions between WT and *cln3* MO morphants confirmed that this difference was statistically significant (*p*<0.0001). The premature death associated with knockdown of *cln3* can therefore be correlated with the reduced longevity of human patients, and the degree of *cln3* knockdown.

### *cln3* morphants display abnormal motor behaviour

Most juvenile CLN3 disease patients experience seizures and myoclonic jerks and lose normal motor function [10]; [16]. Zebrafish embryos first move at 17 hpf, when they develop spontaneous motor contractions causing coiling [34]. We hypothesised that coiling and later tail flicking may change in *cln3* morphants. The number of spontaneous coiling events at 24 hpf and tail flicking at 48 hpf showed no difference between WT and *cln3* ATG MO (1.6 ng) morphants (*p* = 0.4566, 24 hpf and *p* = 0.3306, 48 hpf). However at 36 hpf, a significant difference (*p*<0.0001) was seen, with morphants displaying considerably more tail flicks (Fig 5C–5E). Although movements in *cln3* morphants were more frequent, they displayed weaker muscular contractions—morphants were only able to weakly flick their tails, whereas WT tail flicks caused the embryo to rotate within the chorion (compare S1 and S2 videos).

By 48 hpf, a touch to the end of the tail of a zebrafish typically results in movement away from the source [35]. We determined the response of *cln3* ATG MO morphants to this touch response test at 4 dpf. WT larvae responded very robustly to stimulus by forming a C-bend and darting very quickly away (Fig 5F). In contrast, even though *cln3* ATG MO (1.6 ng) morphants were able to respond (though it often took several touches), they were unable to escape at high velocity or perform a C-bend before escaping (Fig 5G). We also performed experiments at lower doses and found that locomotion defects were always accompanied by morphological defects (data not shown). These data support the theory that the loss of CLN3 protein in zebrafish causes motor abnormalities.

### Axonal tracts are aberrantly targeted in *cln3* morphants

**Fig 6:**
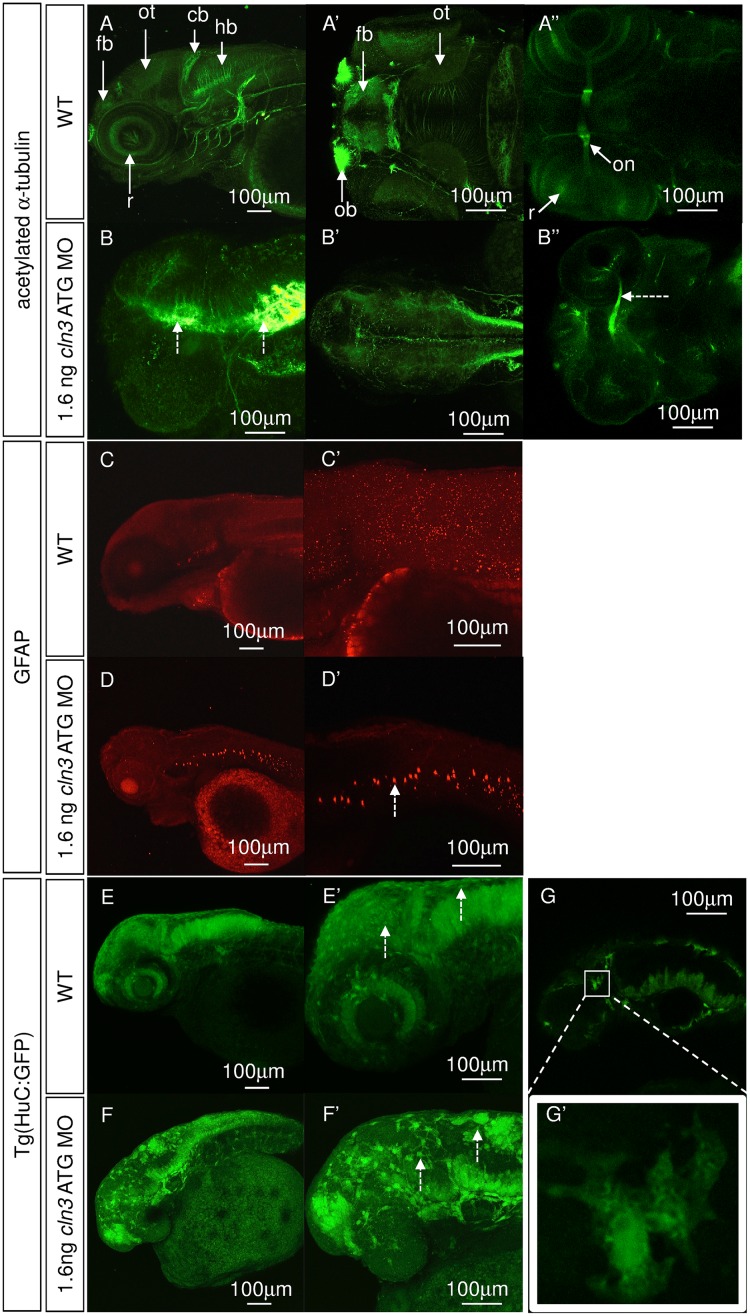
Neurons and glia are disrupted in *cln3* ATG MO morphants. (A-A”, B-B”) Immunohistochemical staining for axons (acetylated α-tubulin) at 4 dpf. (A-A”) normal development of axons in WT larvae. (B-B”) 1.6 ng *cln3* ATG MO morphants have a complete absence of axonal organisation throughout the brain, with axonal accumulation (B, dashed arrows), loss of the optic tectum and a narrowing of the optic nerve (B'', dashed arrow). (A-A”, B-B”), anterior to the left; A, B, lateral view, dorsal up; A’, B’, dorsal view; A”, B” ventral view. (C-C’, D-D’) Immunohistochemistry using antibodies to glia (glial fibrillary acidic protein, GFAP) at 4 dpf. (C-C”) Normal staining in WT larvae. (D-D’) Ectopic GFAP is observed in the notochord in 1.6 ng *cln3* ATG MO morphants (dashed arrow). Lateral view. Anterior to the left. Dorsal up. (E-E’, F-F’, G-G’) Transgenic zebrafish expressing GFP under the control of the *HuC* promoter in neurons were injected with 1.6 ng *cln3* ATG MO and observed at 3 dpf. (E-E’) In WT zebrafish, the normal structure of the developing brain and retina can be observed. (F-F’) In morphants, there appear to be fewer neurons and the normal brain structure is lost. Many GFP-positive cells were enlarged and found nearer the surface of the brain (F’, dashed arrows). (G-G’) When the morphology of these enlarged cells was examined further, they lacked typical neuronal morphology. Lateral view. Anterior to the left. Dorsal up. Abbreviations: cb, cerebellum, fb, forebrain; hb, hindbrain; ot, optic tectum; on, optic nerve. A-G” (all images) Z projection. Scale bars: 100 μm. *n* = 4 per group.

In the zebrafish model of CLN2 disease, axonal tracts are highly disorganised [29] and, although data is not available, this was also reported in the CLN2 disease mouse model [36]. We therefore explored axonal organisation in the *cln3* ATG MO morphants compared with WT using immunofluorescence for acetylated α-tubulin (Fig 6). The results showed that *cln3* ATG MO (1.6 ng) morphants aged 4 dpf had a marked lack of axonal organisation in the brain, that was particularly marked by the loss of axons in the optic tectum and developing cerebellum (Fig 6B and 6B’). It was also noted that the majority of axons seemed to remain near their cell body, rather than extending to their respective targets (Fig 6B). Although an optic nerve was present, it was thinner in comparison with WT, perhaps due to the observed reduction in retinal ganglion cells (Fig 6B”).

### Astrocytosis is present in *cln3* morphants

The activation of astrocytes is a feature of NCL, including juvenile CLN3 disease [17] and is also found in animal models of NCL, and in the case of *Cln3* mutant mice, occurs early in the disease pathogenesis, prior to neuronal death [37, 38]. Immunofluorescence for glial fibrillary acid protein (GFAP), a marker of astrocytes in normal zebrafish and of astrocytosis in reaction to disease, showed GFAP positive cells present throughout the central nervous system in WT and *cln3* ATG MO morphants (1.6 ng) (Fig 6C and 6C’). However, in *cln3* ATG MO morphants, ectopic GFAP, most likely due to astrocytosis, was apparent in the notochord (Fig 6D and 6D’).

### *cln3* morphants display considerable neuropathology

Since we determined that the axonal trajectories of *cln3* ATG MO morphants were abnormal (Fig 6B and 6B’), we next explored the presence of additional neuronal pathology. *cln3* ATG MO (1.6 ng) was injected into transgenic zebrafish embryos expressing GFP under the control of the *HuC* promoter [39], a line used to label neuronal cell bodies (Fig 6E–6G and 6E’–6G’). At 3 dpf we observed that the morphants had an apparent reduction in neuron number and a dramatic loss of order to their distribution (Fig 6F and 6F’), compared with WT (Fig 6E and 6E’). Neuron loss was particularly evident in the midbrain, cerebellum, hindbrain and retina. Many of the labelled cells in morphants were enlarged, peripheral (dashed arrows in Fig 6F’), and lacking typical neuronal morphology (Fig 6G and 6G’). Although similar cells can be seen in WT fish, the cells are less numerous and smaller (dashed arrows in Fig 6E’).

### Cellular proliferation is impaired and retinal area reduced in *cln3* morphants

**Fig 7:**
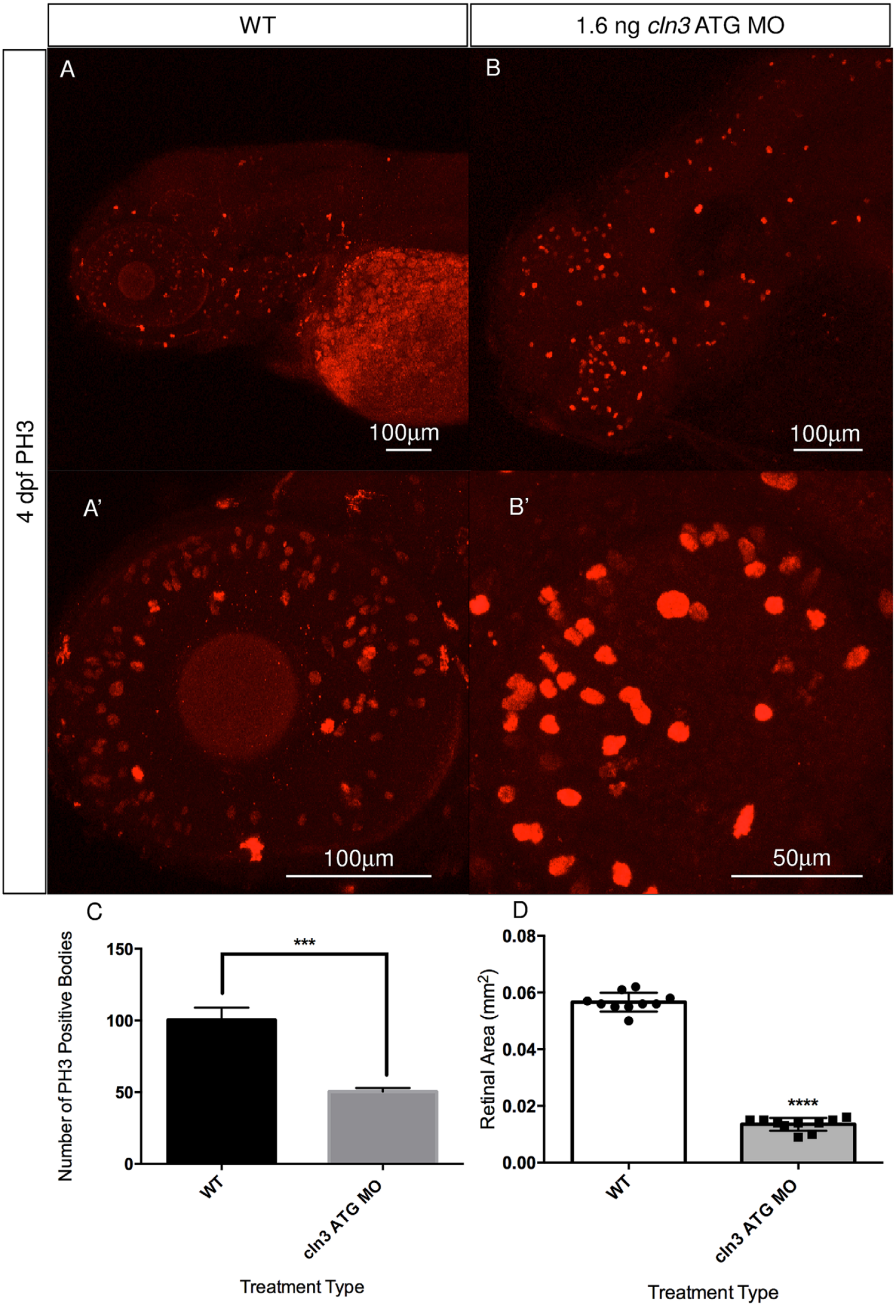
Cellular proliferation is abnormal in *cln3* ATG MO morphant zebrafish. (A, A’) Proliferation, assayed at 4 dpf using anti-PH3 (a marker of proliferative cells in mitotic M phase), is observed throughout the 4 dpf WT retina, jaw and the brain. (B, B’) A marked reduction in the amount of cellular proliferation throughout the retina can be seen in the 1.6 ng *cln3* ATG MO morphant. Although not quantified, it appears that proliferation in the morphant brain (B) is increased compared to WT. Confocal images are Z-projections. Scale bar: 100 μm (A, A’, B) and 50 μm (B’). Lateral views. Anterior is to the left. Dorsal is up. (C) Quantification of these data show that the number of proliferating cells in the morphant retina is significantly reduced from 100.3 cells in WT to 50.3 cells in morphants; ***p<0.0006 (*n* = 3 zebrafish per group). (D) Quantification demonstrating a significantly reduced mean retinal area in the morphants (0.0566 mm^2^ for WT retinae compared to 0.0135 mm^2^ for morphant retinae; ****p<0.0001 (n = 10 zebrafish per group)). (C, D) Data represent mean ±SD; results were evaluated using a 2-tailed unpaired Student’s *t*-test.

To determine if a general deficit in proliferation in the brain and retina, as seen in the zebrafish model of CLN2 disease [29], may also contribute to the *cln3* ATG MO morphant phenotype, immunofluorescence was carried out using an antibody against the mitotic M stage marker, phosphohistone H3 (PH3) [40] at 4 dpf. Analysis of this assay showed that in WT larvae, proliferative cells are located throughout the retina, jaw and to a more limited extent, the brain (Fig 7A and 7A’). However, in *cln3* ATG MO (1.6 ng) morphant larvae, cellular proliferation appears reduced in the eye, and although not quantified, appears to be increased in the brain (compare Fig 7A with 7B and 7A’ with 7B’). To investigate this apparent reduction in retinal proliferation further, we quantified PH3 positive cells in the retinas of 4 dpf WT and *cln3* ATG MO (1.6 ng) morphant larvae (Fig 7C) and observed a significant reduction in cellular proliferation in the morphant retina. The mean number of proliferative cells in morphants was 50% that of WT retinae, a statistically significant difference (*p*<0.0006). These data suggest that impaired neuronal proliferation contributes to the *cln3* ATG MO morphant retinal phenotype.

We next measured the retinal area of *cln3* ATG MO morphants at 4 dpf. These data showed that the retinal area of the *cln3* morphants was significantly diminished by 77% compared to WT (*p*<0.0001) (Fig 7D). These data showed that knockdown of *cln3* significantly reduces retinal area.

### Apoptotic cell death and increased lysosomal storage were identified in the *cln3* morphant brain

**Fig 8:**
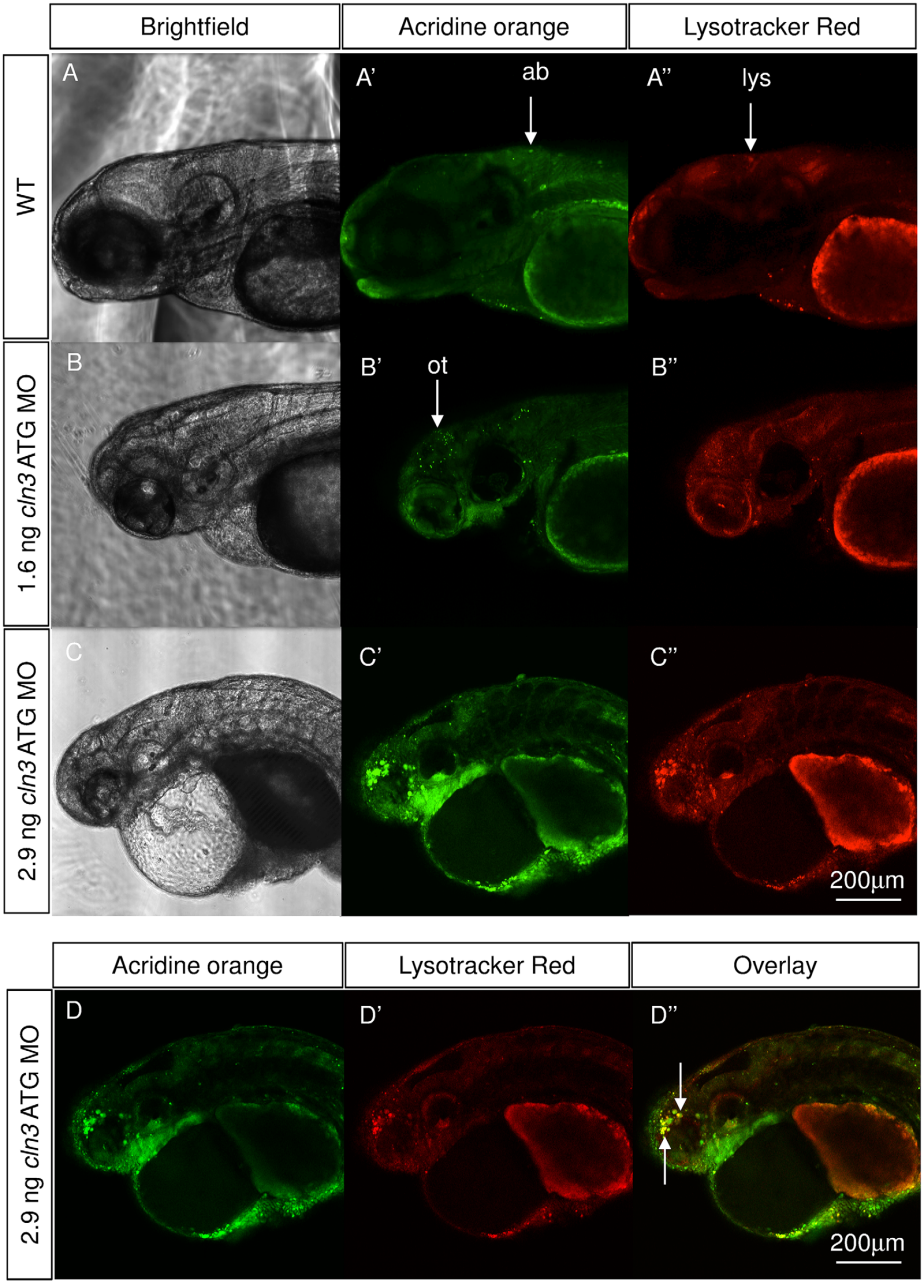
Apoptotic cells and lysosomal storage were found in the *cln3* ATG MO morphant brain. Acridine orange and Lysotracker staining was carried out on live WT and *cln3* ATG MO morphant zebrafish aged 4 dpf. (A-A”) WT fish showed low levels of programmed cell death in the brain and very little bright lysosomal staining. (B-B”) Morphants injected with 1.6 ng of *cln3* ATG MO had increased levels of programmed cell death (acridine orange, bright green) in the brain, this localised particularly to the optic tectum, but only a slight increase in lysosomal staining (red). (C-C”) Morphants injected with 2.9 ng of *cln3* ATG MO showed very high levels of programmed cell death in the forebrain, midbrain, retina and yolk-body boundary; lysosomal hypertrophy was also observed. (D-D”) Morphants injected with 2.9 ng of *cln3* ATG MO exhibit abundant apoptotic bodies in the forebrain that often co-localises with lysosomes (arrows). Bright green, acridine orange in apoptotic bodies; Red, Lysotracker red in lysosomes. Abbreviations: ab, apoptotic body; lys, lysosome; ot, optic tectum. A-A", B-B", C-C” are Z projections. D-D” are the same Z slice. Lateral views. Anterior to the left. Dorsal up. The scale bars represent 200 μm and apply to all panels.

Juvenile CLN3 disease is characterised by loss of neurons, which is particularly marked in the retina, thalamus, cerebral and cerebellar cortices, some of which has been attributed to apoptosis, and also by the lysosomal accumulation of lipopigments [12]; [41]. To examine if programmed cell death and lysosomal storage were present in *cln3* ATG MO morphants, acridine orange and Lysotracker staining was carried out on live larvae prior to fluorescent confocal imaging at 4 dpf. In WT larvae, a small amount of physiological programmed cell death was shown (Fig 8A’) but very few lysosomal puncta were observed at this magnification (Fig 8A”). *cln3* ATG MO (1.6 ng) morphants showed a considerable level of programmed cell death in the brain that localised predominantly to the optic tectum (Fig 8B’), but, as in WT larvae, few lysosomes were brightly stained (Fig 8B”). However, when a larger dose of 2.9 ng *cln3* ATG MO was injected, an even greater amount of programmed cell death occurred in the forebrain, midbrain and retina (Fig 8C’). Abnormal hypertrophy of lysosomes was also observed in larvae injected with the higher dose of morpholino (Fig 8C”) and this was shown to co-localise with acridine orange staining in some regions, particularly in the forebrain (Fig 8D”), which may indicate the phagocytosis of dead neurons.

### Subunit c of the mitochondrial ATP synthase co-localises with lysosomes

**Fig 9:**
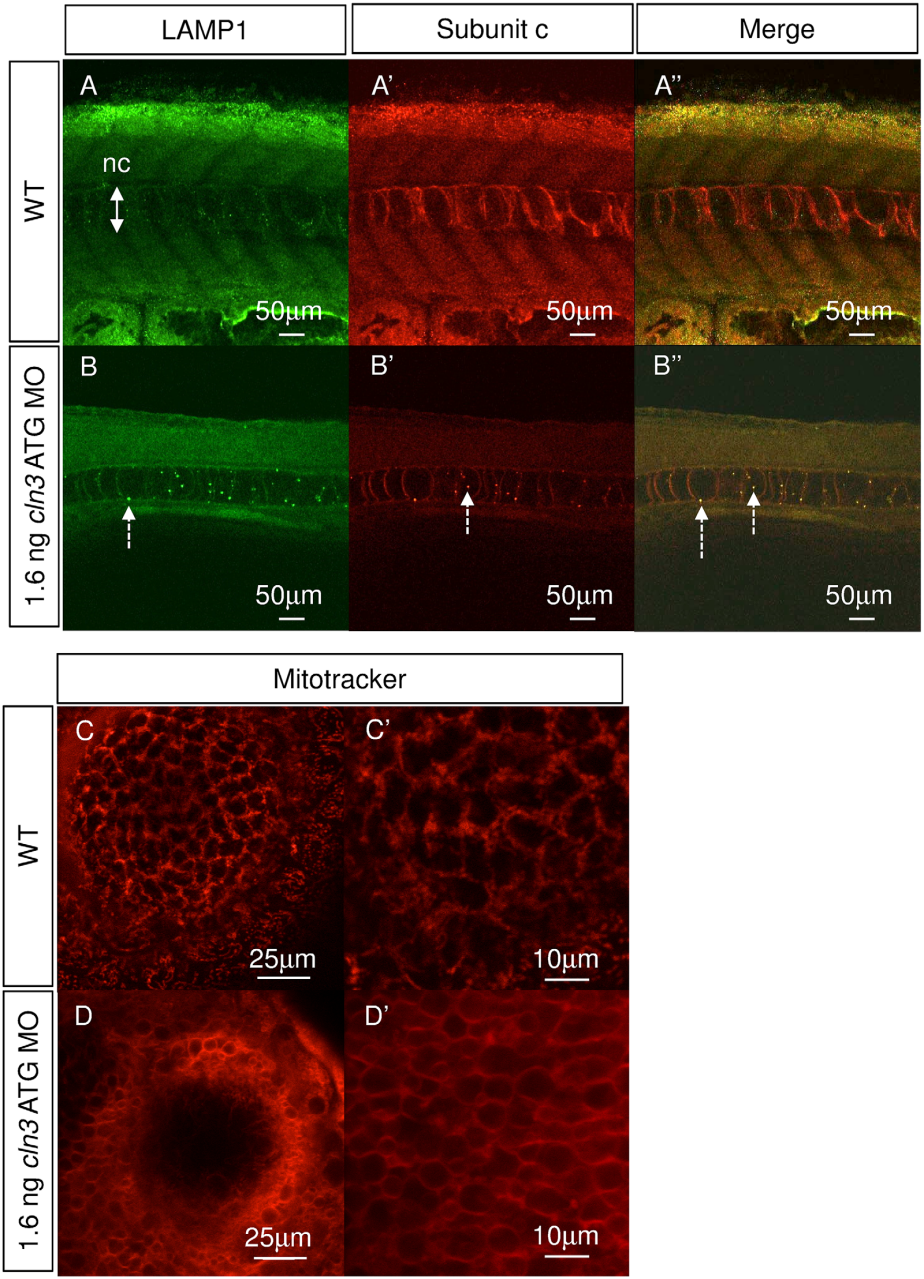
Subunit c accumulates in lysosomes, and mitochondria are compromised in *cln3* ATG MO morphants. (A-A", B-B") Immunohistochemical staining for lysosomal associated membrane protein 1 (LAMP1, green) and subunit c of the mitochondrial ATP synthase (subunit c, red) at 4 dpf. (A-A”) WT. (B-B”) 1.6 ng *cln3* ATG MO morphants. In morphants, lysosomes appear larger (B, dashed arrow), subunit c accumulates (B’, dashed arrow) and they co-localise (B”, dashed arrows). Abbreviations: nc, notochord. Z slice. Lateral view. Anterior to left. Dorsal up. Scale bars: 50 μm. (C-C’, D-D’) Mitotracker stain labelling mitochondria in superficial cells of the eye at 4 dpf. (C-C’) WT. (D-D’) 1.6 ng *cln3* ATG MO morphant. In WT cells, many individual mitochondria are observed, whereas in morphant cells the stain has not been accumulated by mitochondria suggesting loss of mitochondrial membrane potential. Z slice. Lateral view. Scale bars: C, D 25 μm; C’, D’ 10 μm.

A characteristic component of most of the NCLs, including CLN3 disease, is the lysosomal accumulation of subunit c, which makes up almost 50% of the storage mass (Palmer *et al*., 1995). To test if *cln3* ATG morphants accumulate subunit c, immunofluorescence was carried out on 4 dpf larvae using antibodies against subunit c and the lysosomal marker lysosomal-associated membrane protein 1 (LAMP1). Although subunit c and LAMP1 are ubiquitously present, co-localisation of these markers in enlarged puncta showed that subunit c accumulates in lysosomes in the *cln3* ATG MO morphant notochord at 4 dpf (Fig 9B and 9B”), but not in WT larvae (Fig 9A and 9A”).

### Mitochondria in *cln3* morphants were abnormal

In several of the NCLs, including CLN3 disease, abnormally enlarged mitochondria and reduced activity of respiratory chain complexes has been reported [42]. Mitochondria were therefore stained in live zebrafish larvae at 4 dpf using Mitotracker Red [43]. In WT larvae (Fig 9C and 9C’) individual mitochondria can be resolved, some of which are rounded and some elongated. In *cln3* ATG MO (1.6 ng) morphants the stain was not efficiently taken up by the mitochondria, but remains diffuse, thus indicating that they have reduced membrane potential (Fig 9D and 9D’).

## Discussion and Conclusion

In this study, we successfully knocked down *cln3* in zebrafish and demonstrated a range of embryonic and larval phenotypes relevant to CLN3 disease: patient signs reproduced include seizures, motor deficits and premature death. The expected pathological changes observed included programmed cell death, astrocytosis, reduced neurons, reduced neuronal projections, lysosomal storage of subunit c and mitochondrial deficits. In relation to expected changes in morphology, we demonstrated reduced brain and retinal tissue, expanded brain ventricles, and cardiac defects. These combined features confirm that zebrafish *cln3* morphants do indeed recapitulate many features of CLN3 disease, despite occurring during early development and progressing very fast. In patients, onset is juvenile and progression is slow, resulting in many patients living to their second or third decade [2]. Nevertheless, this zebrafish model will be a useful tool for studying this condition as discussed later. In particular, we have documented epileptiform activity, which has not been observed as a natural occurrence in mouse models of CLN3 disease. In two mouse models of CLN3 disease, increased susceptibility to seizure induction has been noted [18] but EEG has not been performed to confirm natural seizure susceptibility. As seizures are a key symptom of the human disease, zebrafish *cln3* morphants may be particularly useful for studying this.

Our data demonstrated an increase in programmed cell death in the brain of *cln3* morphants (1.6 ng MO) when lysosomal storage was not evident, suggesting either that neurons are dying in the absence of storage or that there is a lysosomal acidification defect reducing lysosomal uptake of Lysotracker, as suggested in mouse *CbCln3*^*ΔbCln3*^ cells [44] and patient fibroblasts [45]. However, at a higher dose of morpholino (2.9 ng), increased Lysotracker staining due to lysosome hypertrophy indicates that lysosomal pH is unlikely to be significantly affected. We suggest that the larger lysosomes reside in microglia: as phagocytic cells, microglia are likely to have relatively large lysosomes, and when deficient in *cln3*, they are expected to be even larger. In support of this, we observed GFP from neurons (*HuC*:*GFP*) in cells with morphology that could indicate microglia or phagocytes—perhaps microglia have phagocytosed debris from apoptotic neurons but are unable to degrade the GFP. Indeed, recent evidence supports the hypothesis that the microglial response promotes disease progression in a mouse model of CLN3 disease [46].

Although EEG revealed high amplitude spiking events in *cln3* morphants, reflecting epileptiform activity perhaps similar to that seen in humans [10], we were not able to correlate this with movement as at the stage when we can perform EEG, cln3 morphants are less able to move. The increased tail flicking activity at 36 hpf may be related to seizure or myoclonic activity, as proposed for the zebrafish model of EAST syndrome [32], but could also be due to developmental delay and a prolonging of the earlier spontaneous coiling phenotype. Unfortunately, at this time, it is not possible to perform EEG on 36 hpf zebrafish to confirm seizures. On the other hand it is not possible to carry out EEG on moving fish: the artificial paralysis required to avoid movement artefacts means that spiking events cannot be correlated with ictal (during a seizure) or inter-ictal (not correlating with a seizure) motor activity. A further limitation is that, in contrast to EEG from humans, in which recordings from multiple electrodes are taken, our technique does not allow differentiation between generalised or partial seizures.

The significance of the increased amplitude spiking around 2–4 Hz in *cln3* morphants is unclear, but has also been shown in other zebrafish epilepsy models [32]. Synchronisation in this range (3 Hz) is characteristic of a number of human epilepsies including idiopathic generalised epilepsies (age-related), childhood absence epilepsy (pyknolepsy), juvenile absence epilepsy and epilepsy with myoclonic absences (Commission on Classification and Terminology of the International League Against Epilepsy, 1989). Furthermore, spike and wave epileptic seizures in humans are characterised by 3 Hz oscillations, resulting in sudden-onset generalised seizures and this pattern is also seen in various animal models including cats, rats, mice and monkeys [47]. It may be that this 3 Hz activity is related to inhibitory thalamocortical connections and the involvement of both GABA_A_ and GABA_B_ receptors [47]. We postulate that the EEG results in this study are relevant to human disease and may be used to investigate seizure pathogenesis further and test for anti-epileptic drugs beneficial in CLN3 disease.

We have also demonstrated that the phenotype is progressive in *cln3* morphants, although it is difficult to compare zebrafish and human ages. CLN3 patients lose mobility by their teenage years [16] compared to the severe motor deficit in *cln3* morphants at 4 dpf, when, although able to sense touch, their ability to escape was greatly attenuated. Earlier in development, however, *cln3* morphants were capable of moving, suggesting a progressive loss of motor function. Spontaneous coiling arises from a primitive spinal circuit, as demonstrated by the fact that lesions of the hindbrain have no effect on these behaviours but do affect touch response and swimming [34]. *cln3* morphants are able to sense touch to the tail, although several touches were necessary to elicit a response, indicating that trigeminal neurons and Rohon-Beard neurons are at least partially intact. Both these categories of sensory neurons project to the hindbrain where they synapse with reticulospinal neurons [34]. Together these data suggest that the reticulospinal system is compromised, which is supported by the observation that the hindbrain is smaller than in WT larvae. A marked atrophy of the cerebellum was also observed in *cln3* morphants, and this is likely to contribute to the failure to integrate sensory and motor functions.

In addition to expected phenotypes, this study has revealed reduced proliferation in the retina as a potential contributing factor to the smaller retina in *cln3* morphants, similar to what is seen in zebrafish *tpp1*^*sa0011*^ mutants (model of CLN2 disease; [29]). *cln3* morphants also have a larger yolk sac than WT. Absorption of the yolk depends upon autophagy [48] and larvae with defective autophagy such as *cathepsin D* mutants [49] have large yolks, as do *tpp1*^*sa001*^ mutants [29]. This suggests that autophagy may be altered in zebrafish *cln3* morphants, as in the CLN3 disease mouse model [50], patient-derived iPSCs [51] and patient fibroblasts [52], however we cannot exclude an effect of overall developmental delay.

We propose that this zebrafish model of CLN3 disease may prove particularly useful for finding new small molecule therapies through *in vivo* screening, due to its small size at phenotypic onset which enables more animals to be treated and assayed at a time [28]. Both anti-epileptic drugs and disease-modifying drugs are needed for CLN3 disease. In zebrafish *cln3* morphants, seizures were preceded by an increase in tail flicking, and we suggest that this phenotype could be exploited for compound screening using an automated activity or movement assay [53]. Furthermore, automated fluorescent assays for programmed cell death, lysosomes or microglia could be employed to find other potential therapies [54]. Hence, this new model of CLN3 disease could fill the need for an *in vivo* model suitable for high-throughput screening.

## Supporting Information

S1 Fig Abnormal morphology was observed using two different *cln3* morpholinos at 4 dpf. (A, A’) WT. (B, B’) 1.6 ng *cln3* ATG MO. (C, C’) 12 ng *cln3* SPL MO. Morphant larvae (B, B’, C, C’) showed small retinas, small brain, pericardial oedema, a large yolk sac and abnormal tail curvature (dashed arrows) compared to WT (A, A’). Abbreviations: r, retina; ps, pericardial sac; y, yolk; t, tail; fb, forebrain; mb, midbrain; hb, hindbrain. Lateral views. Anterior to left. Dorsal up. Scale bars: A-C 250 μm; A’-C’ 125 μm.
(TIF)

S1 Video Video of normal coiling activity in 36 hpf WT zebrafish. (MP4)

S2 Video Video of abnormal coiling activity in 1.6 ng *cln3* ATG MO morphant 36 hpf zebrafish, demonstrating weak contractions. (MP4)

## References

[1] Verity C, Winstone AM, Stellitano L, Will R, Nicoll A. The epidemiology of progressive intellectual and neurological deterioration in childhood. Arch Dis Child. 2010;95(5):361–4. 10.1136/adc.2009.173419 . 19948513

[2] Mole SE, Williams RE, Goebel HH. The neuronal ceroid lipofuscinoses (Batten disease). 2nd ed Oxford: Oxford University Press; 2011 xxx, 444 p. p.

[3] Warrier V, Vieira M, Mole SE. Genetic basis and phenotypic correlations of the neuronal ceroid lipofusinoses. Biochim Biophys Acta. 2013;1832(11):1827–30. 10.1016/j.bbadis.2013.03.017 . 23542453

[4] Palmer DN, Bayliss SL, Westlake VJ. Batten disease and the ATP synthase subunit c turnover pathway: raising antibodies to subunit c. Am J Med Genet. 1995;57(2):260–5. 10.1002/ajmg.1320570230 . 7668342

[5] Anderson GW, Goebel HH, Simonati A. Human pathology in NCL. Biochim Biophys Acta. 2013;1832(11):1807–26. 10.1016/j.bbadis.2012.11.014 . 23200925

[6] Carcel-Trullols J, Kovacs AD, Pearce DA. Cell biology of the NCL proteins: What they do and don't do. Biochim Biophys Acta. 2015;1852(10 Pt B):2242–55. 10.1016/j.bbadis.2015.04.027 . 25962910

[7] Shacka JJ. Mouse models of neuronal ceroid lipofuscinoses: useful pre-clinical tools to delineate disease pathophysiology and validate therapeutics. Brain Res Bull. 2012;88(1):43–57. 22502604 10.1016/j.brainresbull.2012.03.003

[8] Haltia M. The neuronal ceroid-lipofuscinoses. J Neuropathol Exp Neurol. 2003;62(1):1–13. . 12528813 10.1093/jnen/62.1.1

[9] Schulz A, Kohlschutter A, Mink J, Simonati A, Williams R. NCL diseases—clinical perspectives. Biochim Biophys Acta. 2013;1832(11):1801–6. 23602993 10.1016/j.bbadis.2013.04.008 PMC4631127

[10] Shahwan A, Farrell M, Delanty N. Progressive myoclonic epilepsies: a review of genetic and therapeutic aspects. Lancet Neurol. 2005;4(4):239–48. 10.1016/S1474-4422(05)70043-0 . 15778103

[11] Aberg L, Liewendahl K, Nikkinen P, Autti T, Rinne JO, Santavuori P. Decreased striatal dopamine transporter density in JNCL patients with parkinsonian symptoms. Neurology. 2000;54(5):1069–74. . 10720276 10.1212/wnl.54.5.1069

[12] Goebel HH. The neuronal ceroid-lipofuscinoses. Semin Pediatr Neurol. 1996;3(4):270–8. . 8969009 10.1016/s1071-9091(96)80031-3

[13] Boustany RM, Alroy J, Kolodny EH. Clinical classification of neuronal ceroid-lipofuscinosis subtypes. Am J Med Genet Suppl. 1988;5:47–58. . 3146329 10.1002/ajmg.1320310608

[14] Ostergaard JR, Rasmussen TB, Molgaard H. Cardiac involvement in juvenile neuronal ceroid lipofuscinosis (Batten disease). Neurology. 2011;76(14):1245–51. 21464428 10.1212/WNL.0b013e31821435bd

[15] Palmer DN, Barry LA, Tyynela J, Cooper JD. NCL disease mechanisms. Biochimica et biophysica acta. 2013;1832(11):1882–93. 10.1016/j.bbadis.2013.05.014 . 23707513

[16] Boustany RM. Neurology of the neuronal ceroid-lipofuscinoses: late infantile and juvenile types. Am J Med Genet. 1992;42(4):533–5. 10.1002/ajmg.1320420421 . 1609833

[17] Tyynela J, Cooper JD, Khan MN, Shemilts SJ, Haltia M. Hippocampal pathology in the human neuronal ceroid-lipofuscinoses: distinct patterns of storage deposition, neurodegeneration and glial activation. Brain Pathol. 2004;14(4):349–57. . 15605981 10.1111/j.1750-3639.2004.tb00077.x PMC8095893

[18] Bond M, Holthaus SMK, Tammen I, Tear G, Russell C. Use of model organisms for the study of neuronal ceroid lipofuscinosis. Biochimica Et Biophysica Acta-Molecular Basis of Disease. 2013;1832(11):1842–65. 10.1016/j.bbadis.2013.01.009. WOS:000323586800007. 23338040

[19] Kuhl TG, Dihanich S, Wong AM, Cooper JD. Regional brain atrophy in mouse models of neuronal ceroid lipofuscinosis: a new rostrocaudal perspective. J Child Neurol. 2013;28(9):1117–22. 10.1177/0883073813494479 . 24014506

[20] Neverman NJ, Best HL, Hofmann SL, Hughes SM. Experimental therapies in the neuronal ceroid lipofuscinoses. Biochim Biophys Acta. 2015;1852(10 Pt B):2292–300. 10.1016/j.bbadis.2015.04.026 . 25957554

[21] Kovacs AD, Pearce DA. Attenuation of AMPA receptor activity improves motor skills in a mouse model of juvenile Batten disease. Exp Neurol. 2008;209(1):288–91. 10.1016/j.expneurol.2007.09.012 17963751 PMC4418195

[22] Kovacs AD, Saje A, Wong A, Szenasi G, Kiricsi P, Szabo E, et al Temporary inhibition of AMPA receptors induces a prolonged improvement of motor performance in a mouse model of juvenile Batten disease. Neuropharmacology. 2011;60(2–3):405–9. 10.1016/j.neuropharm.2010.10.010 20971125 PMC3174473

[23] Kovacs AD, Saje A, Wong A, Ramji S, Cooper JD, Pearce DA. Age-dependent therapeutic effect of memantine in a mouse model of juvenile Batten disease. Neuropharmacology. 2012;63(5):769–75. 10.1016/j.neuropharm.2012.05.040 22683643 PMC3408822

[24] Seehafer SS, Ramirez-Montealegre D, Wong AM, Chan CH, Castaneda J, Horak M, et al Immunosuppression alters disease severity in juvenile Batten disease mice. J Neuroimmunol. 2011;230(1–2):169–72. 10.1016/j.jneuroim.2010.08.024 20937531 PMC3118572

[25] Martin-Jimenez R, Campanella M, Russell C. New zebrafish models of neurodegeneration. Curr Neurol Neurosci Rep. 2015;15(6):33 10.1007/s11910-015-0555-z . 25903297

[26] Wager K, Mahmood F, Russell C. Modelling inborn errors of metabolism in zebrafish. J Inherit Metab Dis. 2014;37(4):483–95. 10.1007/s10545-014-9696-5 . 24797558

[27] Wager K, Russell C. Mitophagy and neurodegeneration: the zebrafish model system. Autophagy. 2013;9(11):1693–709. 10.4161/auto.25082 . 23939015

[28] Mathias JR, Saxena MT, Mumm JS. Advances in zebrafish chemical screening technologies. Future Med Chem. 2012;4(14):1811–22. 10.4155/fmc.12.115 23043478 PMC3566566

[29] Mahmood F, Fu S, Cooke J, Wilson SW, Cooper JD, Russell C. A zebrafish model of CLN2 disease is deficient in tripeptidyl peptidase 1 and displays progressive neurodegeneration accompanied by a reduction in proliferation. Brain. 2013;136:1488–507. 23587805 10.1093/brain/awt043

[30] Mahmood F, Mozere M, Zdebik AA, Stanescu HC, Tobin J, Beales PL, et al Generation and validation of a zebrafish model of EAST (epilepsy, ataxia, sensorineural deafness and tubulopathy) syndrome. Dis Model Mech. 2013;6(3):652–60. 10.1242/dmm.009480 23471908 PMC3634649

[31] Heath RJ, Xavier RJ. Autophagy, immunity and human disease. Curr Opin Gastroenterol. 2009;25(6):512–20. 10.1097/MOG.0b013e32833104f1 19826372 PMC2849745

[32] Zdebik AA, Mahmood F, Stanescu HC, Kleta R, Bockenhauer D, Russell C. Epilepsy in kcnj10 morphant zebrafish assessed with a novel method for long-term EEG recordings. PLoS One. 2013;8(11):e79765 10.1371/journal.pone.0079765 24244558 PMC3828195

[33] Gerety SS, Wilkinson DG. Morpholino artifacts provide pitfalls and reveal a novel role for pro-apoptotic genes in hindbrain boundary development. Dev Biol. 2011;350(2):279–89. 10.1016/j.ydbio.2010.11.030 21145318 PMC3111810

[34] Saint-Amant L, Drapeau P. Time course of the development of motor behaviors in the zebrafish embryo. J Neurobiol. 1998;37(4):622–32. . 9858263 10.1002/(sici)1097-4695(199812)37:4<622::aid-neu10>3.0.co;2-s

[35] Granato M, van Eeden FJ, Schach U, Trowe T, Brand M, Furutani-Seiki M, et al Genes controlling and mediating locomotion behavior of the zebrafish embryo and larva. Development. 1996;123:399–413. . 9007258 10.1242/dev.123.1.399

[36] Sleat DE, Wiseman JA, El-Banna M, Kim KH, Mao Q, Price S, et al A mouse model of classical late-infantile neuronal ceroid lipofuscinosis based on targeted disruption of the CLN2 gene results in a loss of tripeptidyl-peptidase I activity and progressive neurodegeneration. J Neurosci. 2004;24(41):9117–26. 10.1523/JNEUROSCI.2729-04.2004 . 15483130 PMC6730049

[37] Pontikis CC, Cella CV, Parihar N, Lim MJ, Chakrabarti S, Mitchison HM, et al Late onset neurodegeneration in the Cln3-/- mouse model of juvenile neuronal ceroid lipofuscinosis is preceded by low level glial activation. Brain research. 2004;1023(2):231–42. 10.1016/j.brainres.2004.07.030 . 15374749

[38] Pontikis CC, Cotman SL, MacDonald ME, Cooper JD. Thalamocortical neuron loss and localized astrocytosis in the Cln3Deltaex7/8 knock-in mouse model of Batten disease. Neurobiol Dis. 2005;20(3):823–36. 10.1016/j.nbd.2005.05.018 . 16006136

[39] Park HC, Kim CH, Bae YK, Yeo SY, Kim SH, Hong SK, et al Analysis of upstream elements in the HuC promoter leads to the establishment of transgenic zebrafish with fluorescent neurons. Dev Biol. 2000;227(2):279–93. 10.1006/dbio.2000.9898 . 11071755

[40] Hendzel MJ, Wei Y, Mancini MA, Van Hooser A, Ranalli T, Brinkley BR, et al Mitosis-specific phosphorylation of histone H3 initiates primarily within pericentromeric heterochromatin during G2 and spreads in an ordered fashion coincident with mitotic chromosome condensation. Chromosoma. 1997;106(6):348–60. . 9362543 10.1007/s004120050256

[41] Persaud-Sawin DA, Boustany RM. Cell death pathways in juvenile Batten disease. Apoptosis. 2005;10(5):973–85. 10.1007/s10495-005-0733-6 . 16151633

[42] Jolly RD, Brown S, Das AM, Walkley SU. Mitochondrial dysfunction in the neuronal ceroid-lipofuscinoses (Batten disease). Neurochem Int. 2002;40(6):565–71. 11850114 10.1016/s0197-0186(01)00128-0

[43] Sallinen V, Kolehmainen J, Priyadarshini M, Toleikyte G, Chen YC, Panula P. Dopaminergic cell damage and vulnerability to MPTP in Pink1 knockdown zebrafish. Neurobiol Dis. 2010;40(1):93–101. 20600915 10.1016/j.nbd.2010.06.001

[44] Fossale E, Wolf P, Espinola JA, Lubicz-Nawrocka T, Teed AM, Gao H, et al Membrane trafficking and mitochondrial abnormalities precede subunit c deposition in a cerebellar cell model of juvenile neuronal ceroid lipofuscinosis. BMC Neurosci. 2004;5:57 10.1186/1471-2202-5-57 15588329 PMC539297

[45] Holopainen JM, Saarikoski J, Kinnunen PK, Jarvela I. Elevated lysosomal pH in neuronal ceroid lipofuscinoses (NCLs). Eur J Biochem. 2001;268(22):5851–6. 11722572 10.1046/j.0014-2956.2001.02530.x

[46] Groh J, Ribechini E, Stadler D, Schilling T, Lutz MB, Martini R. Sialoadhesin promotes neuroinflammation-related disease progression in two mouse models of CLN disease. Glia. 2016;64(5):792–809. 10.1002/glia.22962 . 26775238

[47] Destexhe A, McCormick DA, Sejnowski TJ. Thalamic and thalamocortical mechanisms underlying 3 Hz spike-and-wave discharges. Prog Brain Res. 1999;121:289–307. . 10551033 10.1016/s0079-6123(08)63080-0

[48] He C, Bartholomew CR, Zhou W, Klionsky DJ. Assaying autophagic activity in transgenic GFP-Lc3 and GFP-Gabarap zebrafish embryos. Autophagy. 2009;5(4):520–6. 19221467 10.4161/auto.5.4.7768 PMC2754832

[49] Follo C, Ozzano M, Mugoni V, Castino R, Santoro M, Isidoro C. Knock-down of cathepsin D affects the retinal pigment epithelium, impairs swim-bladder ontogenesis and causes premature death in zebrafish. PLoS One. 2011;6(7):e21908 10.1371/journal.pone.0021908 21747967 PMC3128622

[50] Mitchison HM, Lim MJ, Cooper JD. Selectivity and types of cell death in the neuronal ceroid lipofuscinoses. Brain Pathol. 2004;14(1):86–96. . 14997941 10.1111/j.1750-3639.2004.tb00502.x PMC8095993

[51] Lojewski X, Staropoli JF, Biswas-Legrand S, Simas AM, Haliw L, Selig MK, et al Human iPSC models of neuronal ceroid lipofuscinosis capture distinct effects of TPP1 and CLN3 mutations on the endocytic pathway. Hum Mol Genet. 2014;23(8):2005–22. 10.1093/hmg/ddt596 24271013 PMC3959814

[52] Vidal-Donet JM, Carcel-Trullols J, Casanova B, Aguado C, Knecht E. Alterations in ROS activity and lysosomal pH account for distinct patterns of macroautophagy in LINCL and JNCL fibroblasts. PLoS One. 2013;8(2):e55526 10.1371/journal.pone.0055526 23408996 PMC3567113

[53] Kokel D, Bryan J, Laggner C, White R, Cheung CY, Mateus R, et al Rapid behavior-based identification of neuroactive small molecules in the zebrafish. Nat Chem Biol. 2010;6(3):231–7. 20081854 10.1038/nchembio.307 PMC2834185

[54] Wittmann C, Reischl M, Shah AH, Mikut R, Liebel U, Grabher C. Facilitating drug discovery: an automated high-content inflammation assay in zebrafish. J Vis Exp. 2012;(65):e4203 10.3791/4203 22825322 PMC3476412

