# Supplementary figures and images for: Neurodegeneration and Epilepsy in a Zebrafish Model of CLN3 Disease (Batten Disease)
Source: PLoS One. 2016 Jun 21;11(6):e0157365. doi: 10.1371/journal.pone.0157365 (PMC4915684; doi:10.1371/journal.pone.0157365)

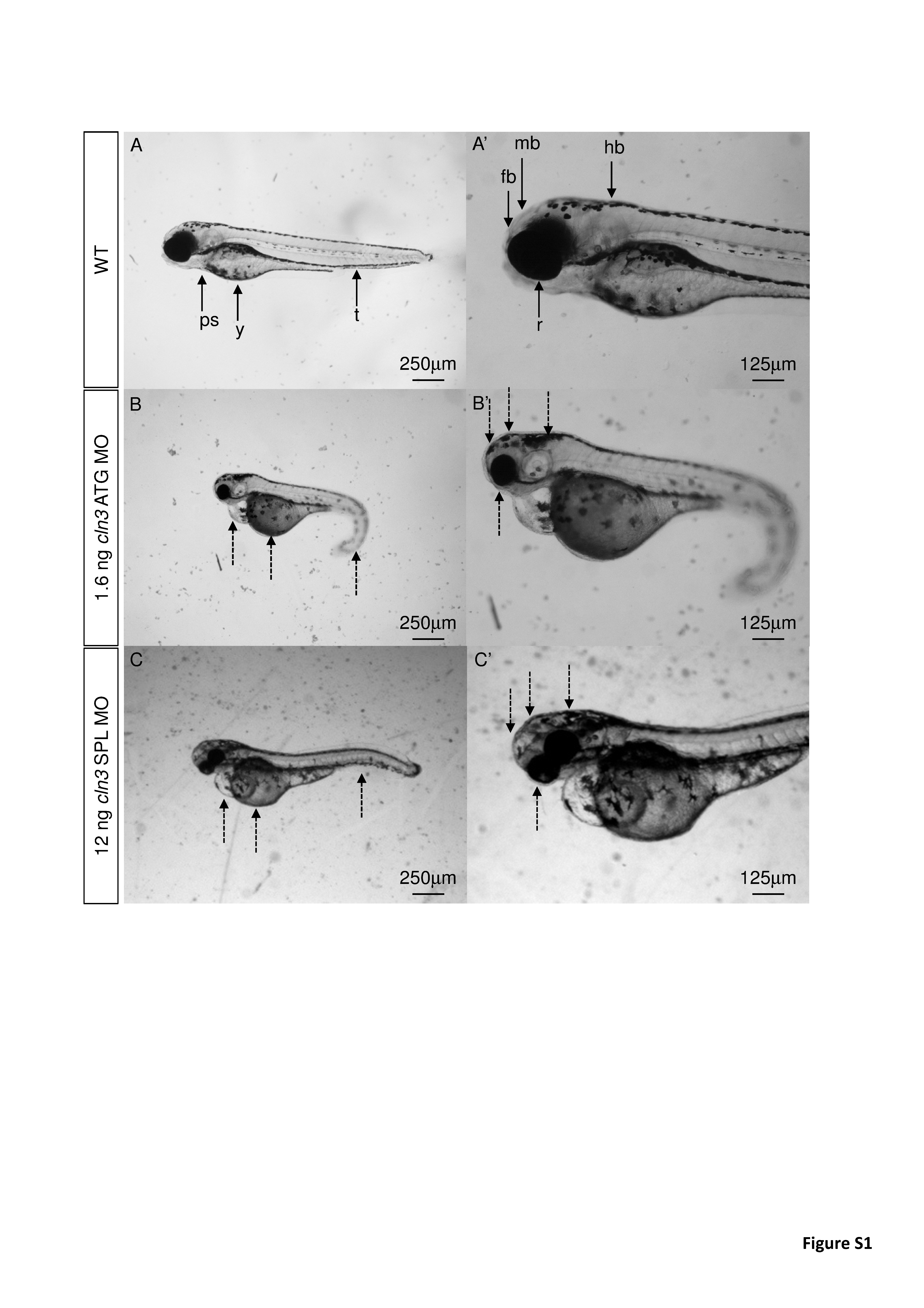

Supplement: S1 Fig — (A, A’) WT. (B, B’) 1.6 ng cln3 ATG MO. (C, C’) 12 ng cln3 SPL MO. Morphant larvae (B, B’, C, C’) showed small retinas, small brain, pericardial oedema, a large yolk sac and abnormal tail curvature (dashed arrows) compared to WT (A, A’). Abbreviations: r, retina; ps, pericardial sac; y, yolk; t, tail; fb, forebrain; mb, midbrain; hb, hindbrain. Lateral views. Anterior to left. Dorsal up. Scale bars: A-C 250 μm; A’-C’ 125 μm. (TIF) [file pone.0157365.s001.tif]
